# Interventions to support spirituality among adults with cancer: a scoping review

**DOI:** 10.1007/s00520-025-09787-x

**Published:** 2025-08-02

**Authors:** Megan Miller, Molly Meyers, Kelly Krainak, Stephen P. Lewis

**Affiliations:** 1 School of Nursing, University of Wisconsin-Madison, Madison, WI USA; 2 Spiritual Care Services, UC San Diego Health, San Diego, CA USA

**Keywords:** Spirituality, Oncology, Supportive care, Spiritual care, Serious illness

## Abstract

**Purpose:**

Spirituality is a core component of holistic cancer care, yet additional support is needed to understand and implement spirituality-focused interventions in practice. The aim of this review was to identify available interventions to address spirituality among people with cancer, to explore common components, and to examine efficacy across interventions.

**Methods:**

A scoping review was conducted. Research questions and criteria were formulated at the outset, followed by identifying relevant publications, charting data, and collating results. Upon identification of available interventions, each was examined for its components and efficacy.

**Results:**

*N* = 26 publications were included, representing *N* = 21 unique interventions. While each intervention varied, they often included key components of prayer, mindfulness/meditation practices, and facilitated sessions with trained spiritual and/or palliative care providers. The effects of interventions varied, with some studies reporting positive outcomes and others reporting mixed effects or no significant changes**.** Notably, individually focused spiritual support interventions were found to increase hope, spiritual well-being, meaning, self-transcendence, and faith; spiritual group therapy interventions were found to increase spiritual health and spiritual well-being (meaning, peace, and faith); mindfulness-based cancer recovery groups were found to increase spiritual well-being; and psilocybin-assisted therapy yielded improvements in spiritual well-being, faith, and connection.

**Conclusions:**

This review offers a novel examination of interventions focused on enhancing spirituality in cancer care. Given spirituality’s central role among many patients and the well-documented desire for spiritual support, future research should clarify which interventions are most effective and under what conditions, to support translation of high-quality spiritual care interventions into practice.

**Supplementary Information:**

The online version contains supplementary material available at 10.1007/s00520-025-09787-x.

## Introduction

Tending to spirituality has been identified as a core component of palliative and supportive care [1]. Various clinical guidelines highlight the importance of addressing spirituality in supportive oncology practice, with calls for regular spiritual care provision to patients and families [2–4]. While definitions of spiritual care vary, this term generally refers to care for the human spirit when facing trauma or difficulty, such as in cancer or other serious illnesses, which can include presence and listening, and addressing needs for meaning, self-worth, self-expression, faith support, and spiritual practices [5, 6]. The process of spiritual care often involves the key elements of screening and assessment, compassionate presence and active listening, engaging in spiritually centered interventions, and collaborating with spiritual care professionals [2, 7, 8].

Attending to spirituality is an important dimension of whole person care [1, 9]. For many individuals, a cancer diagnosis initiates not only a physical and emotional journey, but also a profound spiritual one—prompting questions about meaning, purpose, identity, and hope [10, 11]. Research consistently shows that spirituality is a central coping resource during the cancer experience, linked to improved quality of life, lower psychological distress, and greater resilience across the illness trajectory [12–14]. Although public expressions of spirituality such as religious service attendance have decreased, interest in other aspects of meaning, purpose, and transcendence are increasingly being recognized as important determinants of health with public health implications [15, 16]. In the context of serious illness (such as cancer), patients often identify aspects of religion and spirituality as important along with themes including spiritual coping, spiritual practices, faith beliefs, spiritual transformation, and faith communities [17, 18]. Adults with cancer consistently express the presence of spiritual needs and openness to spiritual care [15, 19], but oncology providers (especially spiritual care generalists, such as nurses and social workers) need more support and training to feel confident to address spiritual care in cancer care settings [20].

Evidence reflects that spiritual care can be an effective means to address psychosocial and physical symptoms, and to improve end-of-life outcomes among people with cancer [21, 22]. A recent systematic review concluded that spirituality is important for many patients with serious illness (such as cancer), spiritual needs are common in palliative settings, spiritual care is frequently desired by patients with serious illness, and spiritual care provision is often associated with improved clinical outcomes [15]. Higher spirituality has also been associated with improved cancer-related symptom outcomes [14, 23] and has been identified as a key element of person-centered care [15]. Additionally, a 2024 systematic review and meta-analysis across randomized controlled trials (RCTs) of spiritual interventions among people with cancer highlighted overall beneficial effects on fatigue, pain, anxiety, depression, and psychological distress, faith, meaning of life, and spiritual well-being [24]. However, this prior review focused only on RCTs and included both spiritual and non-spiritual outcomes, leaving open questions about the broader landscape of spirituality-focused interventions among people with cancer and how they are conceptualized, delivered, and evaluated across diverse research approaches.

Furthermore, even as evidence for the benefits of spiritual care grows, major gaps remain. Patients with serious illnesses (such as cancer) report that spiritual needs are frequently unaddressed, spiritual care is infrequently integrated into clinical settings, and lack of attention to spirituality has been associated with poorer quality of life [15]. Given the centrality of spirituality to the lived experience of cancer and its potential to support quality of life, there is a clear need to better understand the current evidence base around interventions to support spirituality among people with cancer, with a focus on supporting real-world application. Therefore, the purpose of this review is to identify interventions aimed at enhancing spirituality among adults with cancer, to describe those interventions, and to explore the effects of those interventions on spirituality. This review can be used by researchers hoping to advance the science around spirituality-focused interventions in the context of oncology supportive care, and can be used by clinicians as a starting place to begin exploring implementation of interventions to support spiritual care in practice.

## Methods

A scoping review was conducted with multiple searches from August 2023 through July 2024. Scoping review methodology was selected due to the desire to map an initial understanding of the evidence around a broad set of interventions to address spirituality using qualitative, quantitative, and mixed methods. Scoping reviews are ideal for areas of inquiry which focus on broadly understanding an area of evidence, rather than seeking to provide clinical guidance, and they are unique in that they allow for inclusion of publications using various methodologies [25, 26]. This inclusive approach is a strength of scoping reviews, as it enables examination of not only intervention efficacy (as assessed in quantitative studies), but also rich contextual and experiential insights that are often captured in qualitative and descriptive work. By including diverse study designs, this review offers a more comprehensive understanding of how spirituality-focused interventions are conceptualized, implemented, and experienced in cancer care. The current review followed the methodological framework laid out by Arksey and O’Malley [27] which includes five main stages: (1) defining the research questions and eligibility criteria; (2) identifying relevant publications; (3) selecting publications; (4) charting the data; and (5) collating, summarizing, and reporting the results. Although these stages are presented in an order, the actual scoping review process is iterative and often involves ongoing searches with adjustments, as needed, throughout the process. A modified version of the Preferred Reporting Items for Systematic Reviews and Meta-Analyses (PRISMA) flow diagram was used [28], along with the PRISMA-extension for Scoping Reviews (PRISMA-ScR) checklist [26] to enhance rigor and aid in reporting.

- Stage 1: Defining research questions and eligibility criteria

Our research questions were as follows:

1. Which interventions are available to address spirituality among people with cancer?
2. What are the components of the available interventions to address spirituality among people with cancer?
3. What are the effects of the available interventions on spirituality among people with cancer?

Inclusion criteria were as follows: (1) publications that reported on an intervention; (2) spirituality was assessed using qualitative, quantitative, or mixed methods (could be included as a primary, secondary, or exploratory outcome); (3) the sample was entirely made up of adults with cancer (any type or stage); and (4) publications were written in English. Publications were excluded if they were (1) published > 10 years ago or (2) non-primary research (reviews, opinion pieces, etc.). We limited our search to studies published within the past 10 years to ensure the inclusion of interventions reflective of current spiritual care practices. We believe that this timeframe allowed us to capture a wide variety of the most contemporary and potentially implementable interventions while maintaining relevance to current clinical contexts. Only primary research was included in an attempt to avoid duplicating evidence.

- Stage 2: Identifying relevant publications

Searches were conducted within PsychINFO, PubMed, Cumulative Index to Nursing and Allied Health Literature (CINAHL), and SocINDEX with the assistance of a health sciences librarian. Initial search criteria were established at the outset of the review. Then, an iterative process was used, with new terms and search combinations being added throughout the searches. Consistent terms were applied across databases, also using subject headings from the US National Library of Medicine’s Medical Subject Headings (MeSH). See Table 1 for example search strategies. Aligned with review criteria, searches were restricted to the last 10 years. Manual searching was also done through key journals and reviewing manuscript reference lists to identify relevant publications.

- Stage 3: Selecting publications

**Table 1:** Example search strategy

Population	Cancer OR Oncolog*
Intervention/outcomes	Spiritual* OR Relig* OR Faith OR Belief system OR Meditation OR Prayer *AND* *Increase OR Improve OR Enhance OR Promote*

Initial searches yielded 1117 results. After removing duplicates, 975 records (including title and abstract) were screened for relevance. The process of screening and identifying records was completed by the first and third authors (M.Miller and K.K.) using Covidence, a web-based collaboration platform that streamlines the production of literature reviews. Our team used Covidence to import and organize citations, identify/remove duplicates, screen titles/abstracts, and document each reviewer’s decisions. Full-text articles (*n* = 56) were accessed for further evaluation by the same two authors (M.Miller and K.K.), with consultation from a third team member, as needed. Covidence was also used for full-text review, with both authors entering their assessment, along with exclusion reason(s), as applicable. A clear list of inclusion/exclusion criteria were created and embedded within Covidence to minimize disputes. Disputes on eligibility and associated reason(s) were flagged in Covidence and managed by discussion between authors until consensus was reached.

**Fig. 1:**
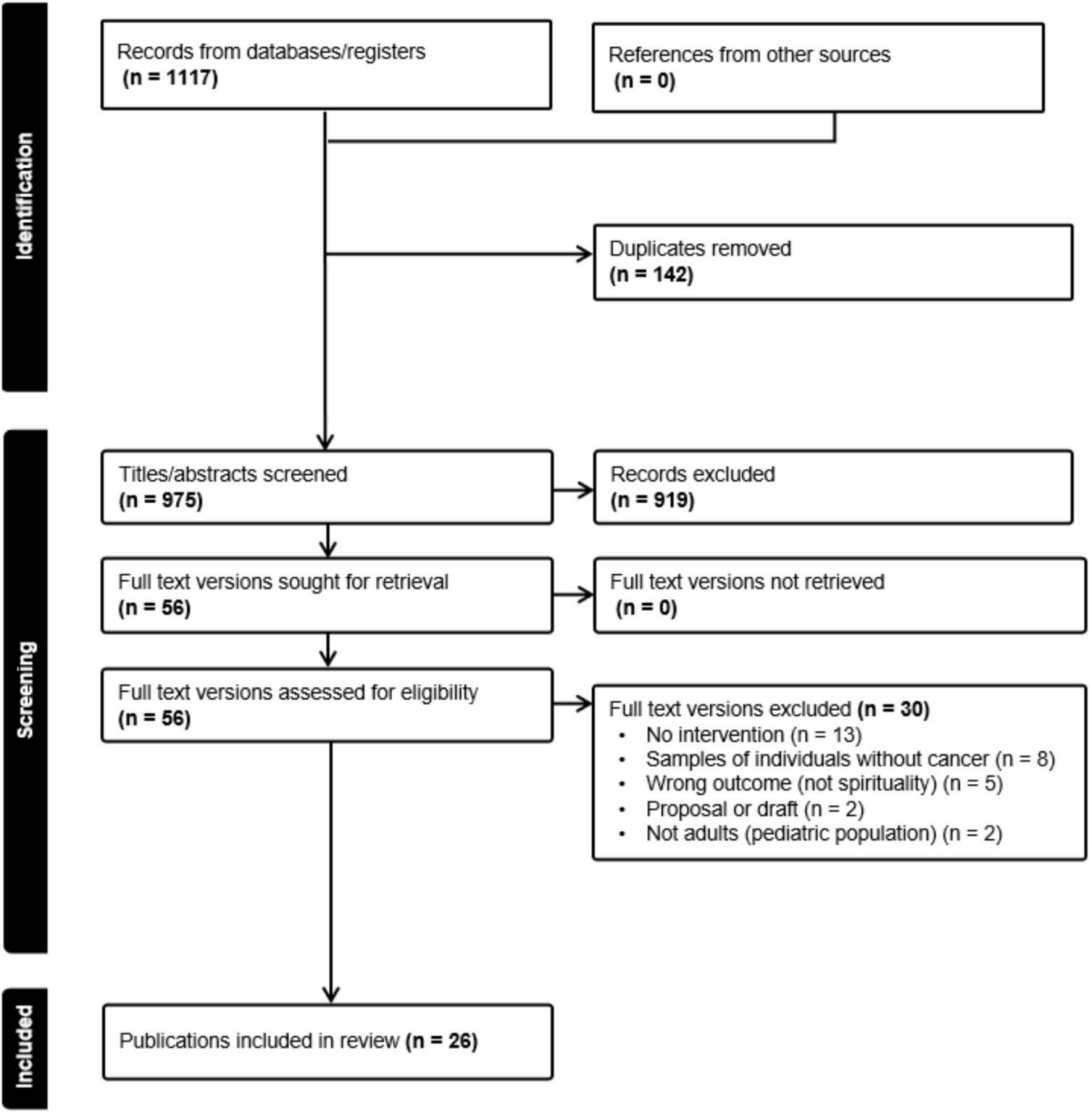
PRISMA flow diagram

Thirty articles were excluded in the full-text review phase. Reasons for exclusion were as follows: no intervention noted (*n* = 13), participants with non-cancer diagnoses (*n* = 8), wrong outcome(s) (*n* = 5), pediatric population (*n* = 2), and proposals or draft reports (*n* = 2). Twenty-six publications were included in the final review (~ 2% of identified citations). See Fig. 1 for a modified PRISMA flow diagram depicting the search and screening process.

- Stage 4: Charting data

Standard information was obtained from each publication using a descriptive-analytical method. Data extraction was completed by the first author (M.Miller) and the second author (M.Meyers). Information was extracted from each publication including (a) author, (b) title of publication, (c) country research was conducted, (d) funding sources, (e) aim of study, (f) study design, (g) inclusion criteria, (h) exclusion criteria, (i) demographic characteristics of patient sample, (j) intervention and comparison examined in study, (k) outcomes assessed, and (l) results. This full table was then edited down to include only key elements. Given that this review aimed to provide an initial mapping of the range of interventions focused on spirituality in the context of adults with cancer, the quality of each publication was not examined. This decision aligns with the standard process of scoping reviews [27].

- Stage 5: Collating, summarizing, and reporting results

## Results

### Overview of included publications

**Fig. 2:**
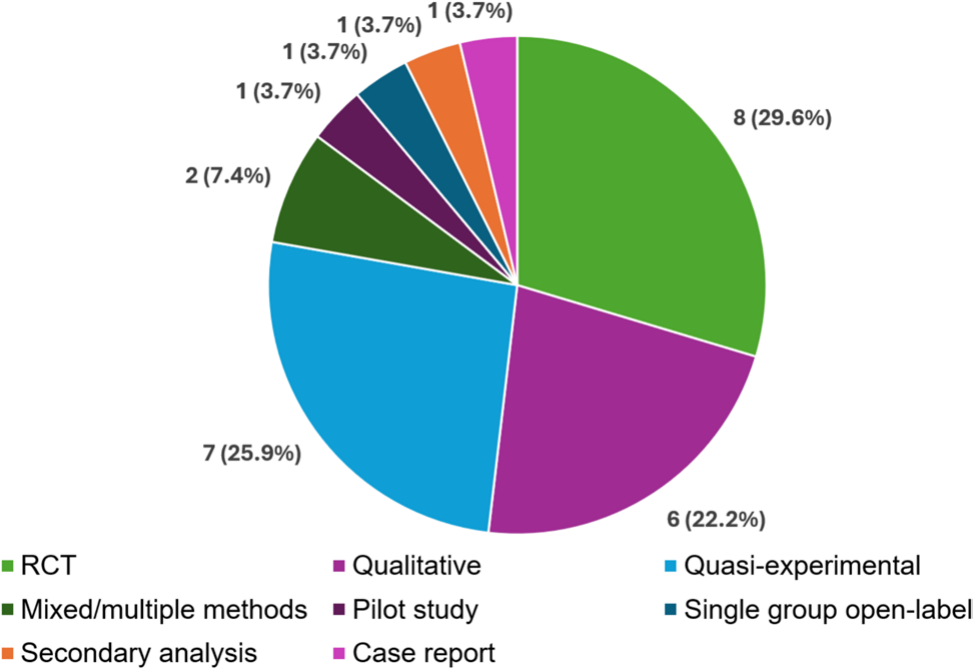
Designs of included studies (*N* = 26)

Publications included in this scoping review spanned reports on RCTs (*n* = 8) [29–36], quasi-experimental studies (*n* = 7) [37–43], qualitative studies (*n* = 6) [44–48], mixed/multiple methods studies (*n* = 2) [49, 50], a pilot study (*n* = 1) [51], a single group open-label trial (*n* = 1) [52], a secondary analysis (*N* = 1) [53], and a case report (*n* = 1) [54] (see Fig. 2). The provided Supplementary Table includes details on each of the included publications. Studies originated from eight different countries, with most from the USA (*n* = 10) [35, 41, 43–46, 48, 51–53], China (*n* = 5) [29, 32, 36, 39, 49], and India (*n* = 4) [40, 42, 47, 54]. Across studies, participants were most commonly white, female (several studies focused specifically on breast cancer [30, 33, 42, 44, 47, 48] and cervical cancer [54]), between the age of 40 and 60 years old, and had at least a high school education.

### Available interventions

Across the 26 included publications, 21 unique interventions were identified within the context of structured research studies [29–45, 49–54], while three additional studies explored the implementation and impact of spiritually focused interventions as they were naturally integrated into real-world settings without a formal research protocol [46–48]. These studies were included to capture how spirituality-related interventions are applied and experienced outside of controlled research environments, contributing to a more comprehensive understanding of current practices. Interventions were considered “unique” if they differed in name and core components (such as delivery method, contents, and/or therapeutic approach). Our team reviewed the descriptions of each intervention in detail to determine whether they represented a distinct intervention model. Interventions included in this review covered a span of approaches, such as a mind map-based life review program [29], spiritual group therapy [37], mindfulness-based art therapy [42], psilocybin-assisted therapy [52, 53], and family-oriented dignity therapy and traditional healing practices [37, 40, 46, 47]. Some interventions were rooted in specific religious traditions [37, 40, 46, 47], while the majority were spiritual but non-religious in orientation [29–36, 38, 39, 41–44, 49–54].

### Components of available interventions

While each of the 21 interventions were unique, they often included key aspects of prayer [30, 37, 41, 46, 47, 54], mindfulness and/or meditation practices [30, 33, 34, 36–38, 42, 48, 50], and facilitated sessions with trained spiritual and/or palliative care providers [31, 36, 40, 43, 45, 50]. Some interventions were guided by established theoretical or conceptual frameworks, while others did not specify such foundations. For example, mindfulness-based interventions were often grounded in Kabat-Zinn’s theory of mindfulness [34, 38], Dignity Therapy interventions drew from Chochinov’s Dignity Model [32, 49, 51], and Spiritual AIM was based on the Spiritual AIM model of spiritual assessment [43]. Other interventions referenced theoretical foundations such as Daoist philosophy, traditional Chinese medicine, and Western psychotherapy models [36]. However, several studies did not clearly refer to a guiding framework. This variability in theoretical grounding may influence the consistency and replicability of findings across interventions.

Interventions were predominantly delivered in-person [29–33, 36, 37, 40–44, 49–54] and in individual settings [29, 31, 39, 41, 43, 51, 53, 54]. Intervention “dosing” ranged, consisting of approximately 4–7 sessions on a weekly or biweekly basis, and around 1–2 h on average per session [29–32, 35–37, 39, 42–44, 49, 51]. Across publications, interventions were delivered by a range of different providers. For example, trained facilitators included spiritual care specialists (chaplains or spiritual healers) [30, 40, 43, 47], as well as spiritual care generalists such as nurses, advanced practice nurses, physicians, trained spiritual care advocates, and counselors [29, 31, 32, 34–41, 49–53]. Across studies, five interventions involved both patient and family/caregiver participation [32, 35, 36, 45, 49], eight delivered spiritual care interventions in group settings [30, 33, 34, 36–38, 44, 52], and three delivered interventions using a virtual or online format [34, 38, 39]. Five studies involved variations of one-on-one support, including visits with chaplains (*n* = 1) [43] and other trained spiritual care generalist providers (*n* = 4) [31, 33, 52, 53]. Across structured interventional studies, intervention sessions often included life review practices (*n* = 4) [29, 31, 39, 51], mind–body exercises (aligned with the National Cancer Institute definition [55]) (*n* = 9) [30, 33, 34, 36–38, 42, 48, 50], and structured prayer practices (*n* = 3) [30, 37, 41]. While most interventions were non-pharmacological, two studies explored psilocybin-assisted therapy, an intervention that involves supported sessions with a classical psychedelic substance [52, 53]. Across studies, control groups were generally provided with usual care, including standard management and education, while one study compared a specialized integrative body-mind-spirit intervention modality to cognitive behavioral therapy [36].

### Effects of interventions on spirituality

Outcomes across studies generally focused on quantitatively measuring changes in spirituality related to the provided interventions. Key instruments used across studies included the Functional Assessment of Chronic Illness Therapy-Spiritual Well-Being (FACIT-SP-12) (*n* = 13) [30–32, 34, 35, 38, 40–43, 49, 51, 53] and the Posttraumatic Growth Index (*n* = 3) [33, 34, 38]. Qualitative and mixed methods studies also explored interventions’ effects on spirituality by asking participants directly about how specific spirituality-focused interventions made a difference in the context of coping with cancer [44, 48, 49]; how religious resources impacted participants when facing cancer and cancer-related symptoms [45, 46]; and more broadly about experiences with cancer [47].

**Fig. 3:**
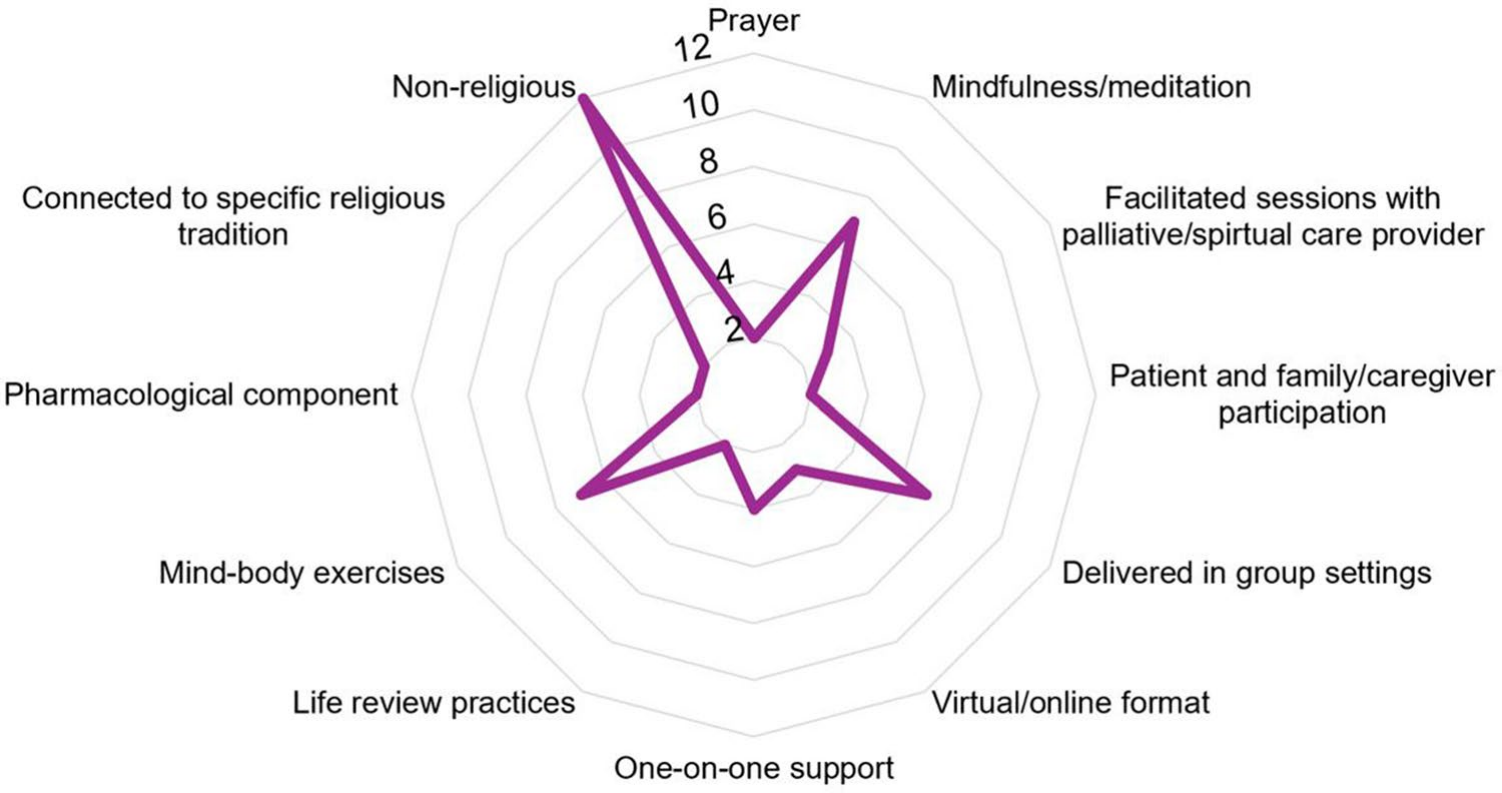
Characteristics of interventions with significant positive effects on spirituality—quantitative (*N* = 14)

Across studies, effects of the included interventions on spirituality varied. Some studies using quantitative methods found significant positive effects related to spirituality, spiritual self-care, or spiritual health and well-being [29, 30, 32–34, 36–40, 42, 43, 52, 53], while other studies did not find significant differences in spirituality-related outcomes [31, 35, 41, 51]. Other studies had mixed findings with significant differences in aspects of spirituality such as faith or self-transcendence, and non-significant trends for religious coping, meaning in life, and hope [39, 43].Various individually focused spiritual support interventions were found to increase hope, spiritual well-being, meaning, self-transcendence, and faith and decrease distress [29, 39, 42, 43, 51]. Spiritual group therapy interventions were found to increase spiritual health and spiritual well-being (meaning, peace, and faith) [30, 37], and mindfulness-based cancer recovery groups were found to increase spiritual well-being and spirituality [34, 38]. Notably, two quantitative studies found that psilocybin-assisted therapy yielded improvements in spiritual well-being, faith, and connection [52, 53], with one of these studies reporting that 96% of participants rated the psilocybin-assisted therapy experience the single or top-five most spiritually significant experience(s) of their lives [53]. Figure 3 provides an overview of the key components of interventions that showed significant positive effects on spirituality. “Significant positive effects” were defined as statistically significant improvements in spirituality-related outcomes (e.g., spiritual well-being, meaning, faith, peace) reported in quantitative studies that tested the efficacy of the intervention. Across the 14 quantitative studies that reported statistically significant improvements in spirituality-related outcomes, the provided figure illustrates some key intervention components, with segment sizes on the radar plot representing how often that component was incorporated (e.g., two interventions involved both patient and caregiver participation, 13 interventions were non-religious).

Qualitative studies found that help from spiritual healers and religious songs were crucial for enhancing spirituality and enduring participants’ experiences with cancer [46, 47]; psychospiritual integrative therapy helped participants embrace the paradox of simultaneously trusting the process of surrender while maintaining control and planning in their lives, fostering new spiritual growth [44]; and Catholic people with cancer found meaning by engaging with spiritual resources, helping others, re-examining priorities, and deepening their faith—shifting their understanding of faith beyond attending mass and saying prayers to strengthening their trust in God despite the pain and suffering caused by cancer [45]. Mixed methods studies found that family-oriented dignity therapy was meaningful, well-received, and yielded increased spiritual well-being [32, 49], while mindfulness-based stress reduction supported participants in reconnecting with their values and spiritual beliefs [50].

## Discussion

This review advances the science of spiritual care by synthesizing evidence around spiritually focused interventions in cancer care and highlighting their potential for clinical translation. These findings underscore the value of integrating spirituality intentionally and systematically into oncology care planning. While heterogeneity in study designs and outcomes limits direct comparison across interventions, the overall evidence points to a meaningful role for spiritual care in enhancing psychosocial outcomes and quality of life during cancer treatment.

Across the 26 included studies, a range of unique interventions were identified and described. The diversity of spirituality-focused interventions uncovered in this review reflects a growing recognition of the need to address spirituality through varied and person-centered approaches within oncology care. Prior research has emphasized that spirituality is a deeply individual experience among people with cancer, shaped by personal beliefs, cultural backgrounds, and illness contexts [56, 57], aligning with a need for a wide range of intervention strategies rather than a one-size-fits-all model. The range of interventions identified, from structured protocols like dignity therapy [32, 51] to real-world applications of traditional and faith-based practices [45, 46], reflects the need for effective spiritual care to be responsive to the diverse backgrounds of people with cancer. Furthermore, the inclusion of “real-world” use of interventions in this review adds valuable insight into how spiritually integrated care unfolds among people with cancer in naturalistic settings, an area often underrepresented in the evidence base. Collectively, findings underscore the evolving landscape and the clinical relevance of spirituality-enhancing interventions within oncology.

The components identified across spirituality-enhancing interventions in this review reflect and extend patterns seen in prior literature. Findings align with previous reviews highlighting mindfulness/meditation, spiritual counseling, and meaning-making interventions as common modalities used to address spiritual needs in oncology and palliative care contexts [1, 24]. Both individual interventions (e.g., family-oriented dignity therapy, spiritual care therapy, psilocybin-assisted therapy) and group-based interventions (e.g., spiritual group therapy, mindfulness-based group programs) demonstrated efficacy, suggesting flexibility across diverse patient needs and care settings. Consistent with previous reviews, variability in theoretical underpinnings and practical approaches may impact the reproducibility and comparative effectiveness of interventions [24, 58, 59]. Furthermore, the use of a wide range of intervention facilitators (e.g., chaplains, nurses) highlights the importance of flexible delivery models that reflect diverse care settings and workforce capacities. Although most interventions were delivered in-person and in individual settings, the emergence of virtual and group-based formats, especially in the wake of COVID-19 [60], points to new opportunities for scalability and access. Together, these findings reinforce the need for greater specification of intervention components and theoretical approaches, alongside efforts to expand training in spiritual care delivery across interdisciplinary oncology teams.

Findings related to the effects of spirituality-enhancing interventions on spirituality build on and enrich current evidence demonstrating that spiritual well-being can be positively influenced through integrative care approaches in oncology. Consistent with prior research [24, 61], this review highlights that a range of interventions, spanning mindfulness-based programs, dignity therapy, and group spiritual counseling, can yield significant improvements in faith, meaning in life, and spiritual well-being among individuals with cancer. The predominance of the FACIT-Sp-12 as a measurement tool [62] aligns with current practices in spiritual care research, offering means to assess and potentially compare outcomes across studies. However, heterogeneity across findings, with some interventions yielding non-significant or mixed results, reflects an ongoing challenge in the field: spiritual outcomes can be complex, multidimensional, and often context-dependent, influenced by cultural background, timing of delivery, and other factors. Qualitative insights further illuminate how individuals experience interventions focused on spiritual support, particularly in the face of existential uncertainty and suffering, which aligns with prior work identifying that grappling with uncertainty is often a key feature of facing cancer [57]. The inclusion of novel modalities such as psilocybin-assisted therapy also underscores a growing interest in innovative, meaning-centered approaches to address existential and spiritual distress [63]. Taken together, results affirm that spiritual outcomes are measurable and meaningfully modifiable through various intervention approaches, reinforcing the importance of integrating spirituality into comprehensive cancer care. Importantly, benefits were observed among patients with varied religious or non-religious backgrounds, indicating that spirituality-focused interventions may support patients in reconnecting with their core values, sources of strength, and inner resilience, regardless of specific faith traditions.

A recent JAMA Delphi expert panel review of 342 studies identified the importance of spirituality to patients experiencing serious illness (such as cancer), noting the effects this has on medical decision-making [15]. The top recommendation of the panel was to include spiritual care as a standard part of patient care [15]. However, there are no currently established clinical guidelines specifically on when and how spiritual care should be provided, contributing to unaddressed spiritual distress [64]. One important pathway to developing these guidelines and delivering competent spiritual care is through the use of generalist-plus-specialist approaches, already common to other areas of medicine such as palliative care [7]. In this model, chaplains function as spiritual care specialists, conducting in-depth spiritual assessments, developing care plans, and collaborating with interprofessional colleagues; while other team members such as nurses, physicians, advanced practice professionals, social workers, and therapists function as spiritual care generalists [7]. Chaplains provide leadership and modeling of spiritual care interventions (such as those identified in this review) offering teachings, storytelling, and encouragement to support generalists in becoming more aware of their own spirituality [65]. Given that multiple interventions in this review were led by non-chaplains, it is clear that interprofessional team members can be highly effective when trained and empowered to participate in spiritual care. For example, clinicians across both inpatient and outpatient settings can incorporate brief screening questions related to spiritual well-being or distress as part of routine symptom assessments. Tools such as the FICA spiritual history instrument (Faith, Importance, Community, Address in Care) provide a structured approach for eliciting patients’ beliefs and preferences regarding the integration of spirituality into their care [66]. When spiritual concerns exceed the scope of generalist providers, referral to chaplains or other spiritual care specialists is appropriate to ensure comprehensive, patient-centered care. Spiritual care interventions offered by generalists rather than specialists may in some cases have a more meaningful impact, in that they communicate to patients that their providers see them as more than simply as the sum of their symptoms or disease processes, and as whole persons [67, 68]. However, significant gaps remain in spiritual care education for healthcare professionals [15, 16].

In order to implement evidence-based spiritual care interventions in cancer care more broadly, it may be useful to focus primarily on those that can be done competently by both spiritual care specialists and generalists. For example, mindfulness-based and meaning-focused interventions are broadly applicable to patients from a variety of religious and spiritual backgrounds and may be more confidently practiced by interprofessional team members [20]. While some effective interventions, such as psilocybin-assisted therapy, may be more time and resource intensive, they should still be considered for intentional integration into oncology practice given the potential for high impact on spiritual well-being and related outcomes [52, 53, 63].

Importantly, the possibility for cultural appropriation of elements of these interventions (or, “when a dominant culture takes from another culture, usually a minority or disadvantaged culture, without full regard for the context, respect, or acknowledgment of the culture it is being borrowed from” [69]), such as Hatha yoga, meditation, and psilocybin-assisted therapy, should be held with deep consideration, intention, and ongoing cultural humility. Exploring and honoring the historical and cultural origins of the interventions, when possible, is recommended [70]. Given the wide range of spiritual and religious traditions in the USA and the well‐established impact of these beliefs on care, regular incorporation of spiritual care interventions may also represent meaningful efforts toward addressing healthcare inequities [15, 57]. For example, Black/African American patients regularly turn to spirituality as a source of coping and support in the context of cancer [71, 72] and often include spirituality as part of serious illness decision-making [73, 74]. Therefore, explicitly including interventions such as spiritual histories and assessments may be particularly useful when working with diverse communities grappling with cancer, as frequently unaddressed cultural and religious factors can emerge and may also lead to important advance care planning conversations [75].

### Limitations

Results from this review must be interpreted within the context of limitations. First, our team made the explicit choice to use scoping review methods rather than systematic review methods. While this choice allowed us to map a broad range of available interventions aimed at enhancing spirituality, both in and outside the context of structured interventions trials, it also limited our ability to draw conclusions about the efficacy of specific interventions. Second, there is potential bias introduced by using search terms focused on positive outcomes (e.g., “increase,” “improve,” “enhance,” “promote”), which may have favored studies reporting beneficial effects. Additionally, the absence of explicit “intervention” terms in the search strategy could have limited the retrieval of some relevant studies. This may have resulted in underrepresentation of null or negative findings. Future reviews should use broader, more neutral search terms to reduce this bias. Third, while our team ran rigorous searches with the support of a Health Sciences Librarian, it is possible that relevant resources were missed. Fourth, spirituality was the central focus of all included interventions, yet definitions and conceptualizations of spirituality varied across studies. Some authors explicitly defined spirituality, while others described it more broadly in terms of meaning, connection, or inner peace, and some did not offer a definition. This conceptual variability reflects diverse cultural and contextual understandings of spirituality, which may influence how interventions are designed, delivered, and experienced [76]. Future research should attend to these cultural nuances and consider standardizing or clearly articulating definitions to support intervention development and cross-study comparison. Fifth, given the varied definitions of spirituality, our team decided to include studies where the effects of interventions on adjacent outcomes were assessed, such as the Post-Traumatic Growth Inventory [77], which contains subcategories across: depth of relationships, interest and expectations in life, discovery of new possibilities and inner personal power, spiritual/religious interest, and appreciation of life.

## Conclusion

This review offers a novel examination of interventions focused on enhancing spirituality in the context of cancer. Interventions range in content, delivery, and efficacy, yet often include common components of interprofessional spiritual care support, life reviews, mind–body practices, and religious practices. Given the central role of spirituality among many patients with cancer and the well-documented desire for spiritual care as part of clinical practice, additional work is needed to examine the efficiency of specific interventions and to support translation of high-quality spiritual care interventions into practice.

## Supplementary Information

Below is the link to the electronic supplementary material.

Supplementary file1 (DOCX 41 KB)

## Data Availability

No datasets were generated or analysed during the current study.
