# Supplementary material for: Interventions to support spirituality among adults with cancer: a scoping review
Source: Support Care Cancer. 2025 Aug 2;33(8):742. doi: 10.1007/s00520-025-09787-x (PMC12317903; doi:10.1007/s00520-025-09787-x)
Supplement: Supplementary file 1 — Supplementary file1 (DOCX 41 KB) [file 520_2025_9787_MOESM1_ESM.docx]

**Article Title:** Interventions to Support Spirituality among Adults with Cancer: A Scoping Review

**Journal Name:** *Supportive Care in Cancer*

**Authors:** Megan Miller, PhD, RN^1^; Molly Meyers, BSN, RN^1^; Kelly Krainak, DNP, APRN, PMHNP-BC^1^; Stephen P. Lewis, MA, DMin^2^

**Institutional Affiliations:**

1. *University of Wisconsin-Madison, School of Nursing, Madison, Wisconsin, USA*
2. *UC San Diego Health, Spiritual Care Services*

**Corresponding Author:**

Name, Degrees: Megan Miller, PhD, RN

Affiliation: UW-Madison School of Nursing

Contact: 701 Highland Ave, Madison, WI 53705, [Miller89@wisc.edu](mailto:Miller89@wisc.edu)

*Supplementary Table 1: Summary of Included Articles*

| *Authors/year* | *Study aim* | *Study design* | *Key sample characteristics* | *Intervention and comparisons* | *Key findings* |
| --- | --- | --- | --- | --- | --- |
| ***(Agarwal et al., 2020)*** | *To explore the spiritual experiences of long-term Brahma Kumaris Raja Yoga meditators diagnosed with cancer to understand how long-term meditation practice influenced their ability to face the physical, emotional, and spiritual challenges of surviving cancer.* | *Qualitative (Interpretative phenomenological)* | *N=3 Ages: 53, 70, 63 Race/Ethnicity: Asian (33.3%), African American (33.3%), and White (33.3%) Sex: Female (100%) Cancer Type: Breast (100%) Stage: 2 (33.3%), 3 (33.3%), 4 (33.3%)* | *Intervention: N/A*  *Control: N/A*  *Qualitative interviews only on Brahma Kumaris Raja Yoga practitioners from meditation centers across the USA.* | *Five super-ordinate themes were identified: Positive state of mind, self-awareness, God's healing power, spiritual support, and spiritual growth. Among the 20 sub-themes that were generated, soul consciousness, awareness of eternity, lack of fear, being happy no matter what, and becoming an inspirational model were new themes that emerged.* |
| ***(Agin-Liebes, et al., 2020)*** | *To determine whether benefits reported at parent study of psilocybin-assisted therapy completion were maintained at two extended long-term follow-up points.* | *Secondary analysis of RCT* | *N=15*  *Mean age 53 (SD=15.5)*  *Race/Ethnicity: White (93.3%) Sex: Female (60%) Male (40%) Diagnosis/Cancer Type: Various, Reproductive (33%) Stage: 1 (33.32%); 2 (20%); 3 (26.67%); 4 (13.33%)* | *Intervention: In the parent study, participants were randomly assigned to one of two groups: psilocybin (0.3 mg/kg) on the first medication session followed by niacin (250 mg) on the second session (i.e. psilocybin-first group), or niacin (250 mg) on the first medication session followed by psilocybin (0.3 mg/kg) on the second session (i.e. niacin-first group). Participants received nine total preparatory psychotherapy sessions and post medication integration sessions delivered by a dyadic therapy team.*  *Control: N/A* | *Significant improvements in spiritual well-being and faith domains (FACIT-Sp-12) at the second long-term follow-up (4.5 years post treatment) relative to baseline. 96% rated the psilocybin experience the single or top five most spiritually significant experience(s) of their lives.* |
| ***(Chen et al., 2020)*** | *To examine the effects of the mind map-based life review program (MBLRP).* | *RCT* | *N=84 Mean Age: 58.99 years (SD= 12.25 years)*  *Sex: Female (39.3%); Male (60.7%) Cancer Type: Gastrointestinal (63.1%), Respiratory (21.4%), Urologic (2.4%), Gynecological (9.5%), Others (3.6%) Stage: Metastasis (88.1%), No metastasis (11.9%)* | *Intervention: Routine Care (health education and pain care) and the MBLRP consisting of four sessions. Each session matched with mind maps, an album, guiding questions, and a video lasting approximately 45-60 minutes. Intervention was conducted at participants' homes over two weeks facilitated by a registered nurse with the MBLRP training.*  *Control: Routine care--health education and pain care).* | *Statistically significant increases in hope and meaning in life were seen at T1 (the second day after completing the MBLRP) and at T2 (four weeks after completing the MBLRP). Statistically significant increases in self-transcendence were seen at T2 (four weeks after completing the MBLRP)* |
| ***(Cipriano-Steffens et al., 2021)*** | *To explore whether increased support for spiritual concerns between the healthcare team and patients through the provision of a Spiritual Care Advocate (SCA) would improve end of life outcomes.* | *Quasi-experimental* | *N=42 patients Mean Age: 60.8, (SD=11.2)*  *Race/Ethnicity: Non-Hispanic White (45%); Non-Hispanic Black (48%); Non-Hispanic Asian (2%); NR (5%) Sex: Female (38%) Male (62%)  Staging: Stage 4 (100%)* | *Intervention: Trained SCAs met with about 20 patients a week (most receiving treatment bi-weekly) for about 20-30 minutes per patient visit. Patient visits were directed by the level of spiritual support patients were comfortable with prayer, readings from scripture, and discussion.*  *Control: N/A* | *Increased spiritual support from the medical community from 27% at baseline to 63% (p=0.005) post SCA intervention, with moderate increases in support between the patient and religious community (55% to 70%, p=0.20), and moderate increases in support between the patient and family (79% to 92%, p=0.30).  Moderate, but not statistically significant increases across quality of life measures for the FACIT-Pal, and benefits, comfort, and coping derived from spirituality in the FACIT-SP.* |
| ***(Dose et al., 2018)*** | *To evaluate patient-reported outcomes of dignity therapy/life plan intervention.* | *Pilot study* | *N=18 total, 50% (n=9) with pancreatic cancer, 50% (n=9) with lung cancer  Of the pancreatic cancer group (n=9): Mean age 63.2 years, (SD= 11.5) Race: White (89%), American Indian (11%) Ethnicity: Other/Non-Hispanic or Latino (100%)*  *Sex: Women (89%), Men (11%)   Of the lung cancer group (n=9): Mean Age 64.0 years, (SD= 13.1) Race: White (100%) Ethnicity: Hispanic or Latino (11%), Other (89%)*  *Sex: Women (22%) Men (78%)* | *Intervention: Dignity therapy/life plan by trained advanced practice nurse including 3 interviews, each 2-3 weeks apart, scheduled to coincide with cancer treatment appointments. First session was a semi-structured audiotaped interview discussing a brief life history, events and people important to them. At the second and third interviews, the first interview was transcribed and edited to reflect subjects that participants wished to be included in a generativity document intended to be given later to family members. At the third session, participants wrote a life plan.*  *Control: N/A* | *No significant changes in measures of quality of life, spirituality, purpose in life, or dignity from baseline to time of Session 3 or at 3-month follow up. Distress levels measured 3 months post-intervention were significantly lower than at baseline.* |
| ***(Hamid & Ahmad Khan, 2021)*** | *To investigate experiences of Kashmiri women with breast cancer regarding social support.* | *Qualitative (phenomenological)* | *N=12 Age: 21-30 years old (33.33%); 31-40 years old (41.67%); 41-50 years old (16.67%), 50+ years old (8.33%) - Race/ethnicity: Kashmiri (100%)*  *- Sex: Female (100%) Diagnosis/Cancer Type: Breast Cancer (100%) - Staging: Stage 1 (33.3%); Stage 2 (41.67%); Stage 3 (25%)* | *Intervention: N/A*  *Control: N/A*  *Qualitative interviews only* | *4 themes from the study: Social support from significant others, important others, health care providers, and religion and spirituality  Regarding Religion and Spirituality:   Help and support from spiritual healers important as women believed spiritual healers had divine qualities to heal people from pain and disease using taweez (amulets), shireen (sugar balls) , animal sacrifice, reading the Quran, and distributing tehri to get rid of disease; however, two of the women had bad experiences with spiritual healers as they felt they were misled.   Visiting shrines in Kashmir to get blessings from God by visiting the saints buried in the shrines.   Offering prayers, making supplications, reading the Quran, helping the needy and poor, and keeping fasts were religious practices that relieved fear and distress.   Received help from the belief that they will be rewarded for their suffering by God.* |
| ***(Hamilton et al., 2016)*** | *To explore how religious songs are used to alleviate cancer symptoms among older African Americans.* | *Qualitative (descriptive)* | *N=31 - Mean age: 62.5 years (SD= 12.4 years) - Race/ethnicity: African American (100%)*  *- Sex: Female (83.9%); Male (16.1%)* | *Intervention: N/A.*  *Control: N/A*  *Qualitative interview only* | *Religious songs used when feeling depressed, low, or sad were: instructive (remind participants of God's love and ability to heal and resolve health issues), thanksgiving and praise, communication (prayers to God), memory of forefathers, and life after death.   Religious songs when feeling weak or seeking strength to endure treatment: thanksgiving and praise, instructive, and communication  Religious songs used in relation to worry, anxiety, and fearfulness: thanksgiving and praise, instructive, ad communication  Religious songs as sources of hope and support: reminders to lean on God and depend on Him for their needs  Religious songs strengthened and enabled survivors to endure adverse effects of chemotherapy and radiation. Reported elevated moods and reduced anxieties, worries, and fears associated with their cancer experience.* |
| ***(Hosseini Rafsanjani et al., 2017)*** | *To evaluate the influence of spiritual group therapy on hope and the mental and spiritual health of patients with colorectal cancer.* | *Quasi-experimental* | *N=64 - Mean age: Intervention (Mean 49.71; SD= 10.76); Control (Mean 45.11; SD=12.10)*  *- Sex: Female (48.4%); Male (51.6%) Diagnosis/Cancer Type: Colorecetal Cancer (100%) - Staging: Stage 1 (9.4%); Stage 2 (31.3%); Stage 3 (59.4%)* | *Intervention: Organized spiritual group therapy sessions for 8 weeks, one 90-minute session weekly with psychological and spiritual interventions and an Islamic approach focusing on meditation, prayer, repentance, altruism, patience and trust.*  *Control: Usual care, meditation CD and booklet only.* | *Intervention group scores of hope, mental health, and spiritual health increased substantially more than the control group.* |
| ***(Jafari et al., 2013)*** | *To investigate the role of spiritual therapy intervention in improving the spiritual well-being and quality of life of Iranian women with breast cancer.* | *RCT* | *N=65 - Mean age: Intervention: 47.9 years, (SD=10.56); Control: 48.1 years, (SD= 10.2) - Race/ethnicity: Iranian (100%) - Sex: Female (100%)Diagnosis/Cancer Type: Breast Cancer (100%)* | *Intervention: Three spiritual healers held six weekly sessions, each with a theme related to domains of spirituality, ending with a 20-30 minute guided relaxation and meditation exercise. Each session lasted ~2-3hr and was a mix of didactic material, Q&A, sharing, reflecting. Main themes/domains of spirituality were: Introduction to spirituality, relaxation and meditation, control, identity, relationships, and prayer therapy.   Control: Standard management and treatment and routine educational program based on nutrition, physical activity, and a radiation therapy patient-education program.* | *Participation in spiritual therapy program was associated with statistically significant improvements seen in spiritual well-being for the intervention group. Significant improvements in meaning, peace, and faith in the spiritual therapy group after intervention. No statistically significant difference was seen in QOL or spiritual wellbeing in control group.* |
| ***(Joshi et al., 2021)*** | *To identify effects of Mindfulness-Based Art Therapy (MBAT) on psychological distress and spiritual wellbeing in breast cancer patients undergoing chemotherapy.* | *Quasi-experimental* | *N=30 - Sex: Female (100%) Diagnosis/Cancer Type: Breast cancer (100%) - Stage: Breast cancer with recurrence (3.33%), Breast cancer with metastasis (3.33%)* | *Intervention: In-person supervised MBAT session by facilitators and then 1 week of daily guided home practice. Sessions included mindfulness meditation for 15 minutes and mindful coloring for 30 minutes daily for 1 week.*  *Control: N/A* | *The total spiritual well-being and median of total meaning, pace, and faith sub-scales significantly increased from pre-session to immediate post-session and from pre-session to 1-week post-session.* |
| ***(Kestenbaum et al., 2017)*** | *To evaluate the feasibility and tolerability of a chaplain-delivered spiritual care intervention using the Spiritual Assessment and Intervention Model (Spiritual AIM) and to evaluate the impact of Spiritual AIM on spiritual well-being, religious and cancer-specific coping, and physical and psychological symptoms.* | *Quasi-experimental* | *N=31 -Mean Age: 59.4 years, (SD= 9.9) -Race/Ethnicity: White (87.1%); Asian (9.7%); Hispanic (3.2%) --Sex: Female (64.5%); Male (35.5%)*  *Diagnosis/Cancer Type: Breast (19%); Gynecological (23%); Gastrointestinal (16%); Prostate (16%); Head/neck (10%); Other (16%)* | *Intervention: 3 sessions with a chaplain for 45-60 minutes (initial and subsequent sessions scheduled 2-3 weeks apart in person or over the phone). Assessed spiritual needs and primary concerns were focused on, then interventions were chosen by chaplains based on each assessment category to inform spiritual care.*  *Control: N/A* | *Post-spiritual AIM, significant increase was observed only in the Faith subscale of the FACIT-Sp-12. There was a trend toward an increase (improvement) in Positive religious coping on the Brief RCOPE, whereas no significant change was seen in Negative religious coping.* |
| ***(Kruizinga et al., 2019)*** | *To evaluate the effect of an interview model for life events and ultimate life goals (Life InSight Application [LISA]) on quality of life and spiritual well-being.* | *RCT* | *N=153 -Mean Age: 62 years, (SD= 10.5) -Race/Ethnicity: Dutch (100%)*  *-Sex: Female (50.3%); Male (49.7%)*  *Diagnosis/Cancer Type: Breast (22.2%); Esophagus (11.1%); Colorectal (23.5%); Brain (6.5%); Gynecological (7.2%); Prostate (9.2%); Gastric (3.9%); Pancreatic (5.2%); Other (11.1%)* | *Intervention: Two 1-hr long consultations at hospital with interview model-trained spiritual counselor. First consultation included discussing important life events and defined life goals. At the second consultation, the review of findings was completed using an iPad application. After the second consultation, patients got a handout with schematic representation of their life events and life goals.*  *Control: Care as usual.* | *Quality of life and spiritual well-being did not significantly change over time between intervention or control groups. The experience of Meaning/Peace significantly influenced quality of life and satisfaction with life.* |
| ***(Lau et al., 2020)*** | *To compare efficacy of* *integrative body-mind-spirit intervention (I-BMS) and cognitive behavioral therapy (CBT) among Chinese patients with lung cancer and their caregivers.* | *RCT* | *N= 157 patient-caregiver dyads  The following data reflects demographics of the patient: - Mean Age: I-BMS 58.6 (SD= 8.5). CBT: 61.4, (SD= 10.3) - Race/ethnicity: Chinese (100%)*  *- Sex: Female (59.9%) Male (40.1%)*  *Diagnosis/Cancer Type: Lung Cancer (100%) - Stage: 4 (63.7%); 3 (19.7%); 2 (6.4%); 1 (2.5%)* | *8-12 participants in a group had 8 weekly 3hr sessions facilitated by 2-3 trained facilitators. Integrative body-mind-spirit intervention (I-BMS) or cognitive behavioral therapy (CBT).  Intervention: I-BMS: psychoeducation about holistic health and practiced mind-body exercises like acupressure and Qigong-inspired movements, mindfulness-based relaxation like meditation. With expressive arts, participated in a life-review for re-constructing meanings out of their cancer or caregiving journey.  Control: CBT group: participants learned relaxation, identified dysfunctional coping patterns/thoughts/values, used a mood diary/reappraisal/cognitive continuum/cost-benefit analysis and positive self-statements for adjusting dysfunctional emotional responses and thoughts. Constructed and implemented plans of pleasurable activities.* | *I-BMS resulted in greater increases in overall quality of life and spiritual self-care than CBT. Both CBT and I-BMS groups experienced improvements in emotional and spiritual domains of quality of life.* |
| ***(Mosher et al., 2018)*** | *To examine whether adding a peer helping component to a coping skills intervention leads to improved meaning in life and peace for advanced gastrointestinal cancer patients and their caregivers.* | *RCT* | *N=50  Patient characteristics: - Mean age: 58.18, (SD= 11.56) - Race/ethnicity: Non-Hispanic white (86%) Missing (2%)  - Sex: Female (38%); Male (62%)*  *Diagnosis/Cancer Type: Colorectal (38%); Pancreatic (26%) Esophageal (12%); Other (24%) - Stage: IV (100%)* | *Peer-helping + coping skills group and coping skills only group completed five weekly 50 to 60 minute telephone sessions with both dyad members participating simultaneously via speakerphone.   Intervention: The Peer-helping + coping skills helped create an information resource on various QOL issues for other patients and caregivers coping with cancer and were told that this resource would be available to others in print and online. Each person was mailed identical handouts that were drafts of the informational resource and a CD with instructions for relaxation exercises. The first four sessions had a similar format. The therapist introduced the session topic (e.g., physical symptoms, stress, social changes) and asked about the dyad's experiences related to the topic. Then the therapist asked the dyad for their advice. Then, the dyad reviewed handouts listing evidence-based coping skills related to the session topic and indicated which skills may be most helpful to others. This was followed by in-session practice of coping skills and/or self-evaluation of their use of coping skills. Then, the dyad indicated whether the skill should be included in the informational resource for others. Finally, each dyad member set a goal for the upcoming week related to the session focus, and goal progress was assessed during the subsequent session. Following the fourth session, the dyad received the handouts with their edits and additions that would be an informational resource for others. During the fifth session, the dyad was asked to critique the handouts further and discuss their use of the skills on the handouts during the past 2 weeks. Then the dyad was asked to provide final advice for others on coping with cancer. Each dyad member was asked to set a goal for the coming week related to one of the session topics.  Control: Coping skills group: Dyads in the coping skills condition discussed the same topics as the PH + coping skills condition, but did not help create an informational resource for other patients and caregivers. The same therapists administered both study conditions. Coping skills participants received the same initial handouts and CD with relaxation exercises and completed the same in-session practice of skills and weekly goal setting. Instead of providing advice to others or critiquing the helpfulness of the skills for others, dyads focused on the helpfulness of the skills for themselves.* | *Intervention was feasible and acceptable for this population. Peer helping in the context of a coping skills intervention did not enhance spiritual well-being relative to coping skills alone. Meaning in life and peace were stable over time for the Peer-Helping + coping skills group, and showed a small increase in the coping skills only group.* |
| ***(Patel et al., 2015)*** | *To qualitatively investigate multicultural breast cancer survivors' experiences of paradox following psycho-spiritual integrative therapy (PSIT).* | *Qualitative (unclear tradition, possibly thematic)* | *N=12 - Mean age: 50.8 - Race/ethnicity: European Caucasian (50%); Hispanic (8.3%); Chinese (16.6%); Indian (8.3%); Eastern European (8.3%); Other (8.3%) - Sex: Female (100%)*  *Diagnosis/Cancer Type: Breast cancer survivors (100%) - Stage: Stage 0 (8.3%); Stage 1 (41.7%); Stage 2 (16.6%); Stage 3 (16.6%); Did not state (16.6%)* | *Intervention: psycho-spiritual integrative therapy [PSIT] - 8-week non-denominational group intervention promoting integration of lived experience of spirituality and the search for existential meaning into psychotherapy. Psychotherapeutic and psycho-educational components included.  Intended for participants to acknowledge and embody paradoxes.*  *Control: N/A* | *Themes identified: attempting to maintain coherence in new and old ways, letting go of ultimate control in life, interconnection between helpers and hinderers, spiritual edges and tensions, and new paths to empowerment.  Spiritual tensions and growth edges: Participants questioned their assumptions and held paradoxical tensions within an expanded sense of consciousness that fostered a change process leading to new spiritual growth edges and enriched experience of existence. Some participants turned to spirituality to cope with breast cancer or experienced spiritual growth by opening to God’s care and larger plan. Some participants held the following paradox: I trust in the surrendering process; I control and plan my life without surrendering. Several participants expressed the understanding that their life events can be surrendered to God, spirit, or the Divine. Some participants found that they had to broaden their spiritual experiences to better cope with their lives after breast cancer treatment.* |
| ***(Poletti et al., 2019)*** | *To explore the impact, feasibility, acceptability, and effectiveness of a Mindfulness-Based Stress Reduction (MBSR) intervention for people with metastatic cancer integrated in Early Palliative Care (EPC).* | *Mixed methods* | *N=20 - Mean age: 54, (SD= 7.72) - Race/ethnicity: Italian (100%)*  *- Sex: Female (85%); Male (15%) - Stage: Stage IV Metastatic Cancer (100%)* | *Intervention: Mindfulness-based stress reduction (MBSR) - 8 meetings for 2.5hr once a week, a 4.5hr session between the 6th and 7th weeks and 0.5hr home practice daily. MBSR included formal sitting meditation, body scan, light yoga, walking meditation, and Aikido exercises. Provided with materials for home practice. A qualified MBSR instructor conducted the program. Sessions were attended by a clinical psychologist and a physician trained in meditation, together with the palliative nurse as facilitators.*  *Control: N/A* | *Participants identified connection with an "abiding inner-centeredness and intimate spaciousness" which was related to a spiritual dimension. Findings also focused on "Opening to spiritual dimensions" which included supporting participants in questioning and reconnecting with their values and spiritual beliefs.* |
| ***(Sankhe et al., 2017)*** | *To evaluate the role of spiritual care in cancer patients and their primary caregivers regarding their spiritual and general well-being.* | *Quasi-experimental* | *N=107 patient-caregiver dyads  Patient characteristics: - Mean age: 51, (SD= 13 years) - Sex: Female (41%); Male (59%)*  *Diagnosis/Cancer Type: Breast (22%); Buccal Mucosa (47%); Tongue (7.5%); Other (23.5%)* | *Intervention: Team including physicians, staff nurses and a spiritual care specialist were involved in delivering spiritual care to patients. Spiritual care was given under MATCH guidelines: Mercy, Austerity, Truthfulness, Cleanliness, Holy name. Spiritual care for all participants was administered for 1.5hr daily (30min each for counseling, reading, and chanting).*  *Control: N/A* | *FACIT-Sp scores were significantly improved at all follow-up time points as compared to baseline.* *Improved level of not only spiritual well-being but general well-being in both the patients and their primary caregivers.* |
| ***(Shinde et al., 2022)*** | *To describe a case involving respite palliative care's role in understanding and managing complex palliative care needs.* | *Case report* | *N=1 - Age: 59 - Sex: Female (100%)*  *Diagnosis/Cancer Type: Cervical cancer (100%) - Stage: 4 (100%)* | *Intervention: Respite Palliative Care (RPC)*  *Control: N/A* | *Analgesics for movement related incident pain which helped her to perform "Namaz" (a daily religious prayer done by Muslims in standing and kneeling or sitting position). Per the report, the interventions offered "seemed fulfilling to the patient." "exploring and addressing patient's spiritual needs further strengthened her trust in HC system."* |
| ***(Shnayder et al., 2023)*** | *To evaluate the changes in NIH-HEALS scores in a cancer population with major depressive disorder undergoing  psilocybin-assisted therapy.* | *Single group open-label trial* | *N=30 - Mean age: 56 years (SD=12, range=30-78) - Race/ethnicity: Caucasian (80%), Black/African American (10%), Asian/Asian American/Pacific Islander (6.7%), Hispanic/Latinx (3.3%)*  *- Sex: Female (70%), male (30%) - Prognosis: Non-curable (53.3%), Curable (46.7%)* | *Intervention: Psilocybin-assisted group therapy - Psychotherapeutic care was provided before, during, and after psilocybin administration to cohorts of 3-4 participants. The therapeutic approach was non-directive and used of eyeshades and a music program to promote an inner-directed experience. The dominant mode of therapy was active listening and presence. Supportive therapy included individual and group prepa­ration sessions, simultaneous administration of psilocybin to the cohort (in adjacent rooms), and individual and group integration sessions. After screening, 7 visits were completed: individual preparation, group preparation, psilocybin session, integration (individual and group components), integration (individual and group components), follow-up x 2..*  *Control: N/A* | *All three factors of the NIH-HEALS (Connection, Reflection & Introspection, and Trust & Acceptance), demonstrated positive change. Effects were apparent one day post-dosing and were sustained up to the last study interval at 8 weeks. The Connection factor, measuring connection to a higher power and to loved ones, increased by 12.7 % on average by week 8 (p = 0.003) the end of the study. Scores on the Reflection & Introspection factor, (sense of meaning, purpose, and gratitude, experience of joy in nature, use of activities that connect mind and body, present moment orientation, and an awareness about the fragility of life) rose by 7.7 % by week 8 (p < 0.001). Similarly, scores on the Trust & Acceptance factor, increased by 22.4 % by week 8 (p < 0.001). Cumulatively, this totaled an average of a 16.4-point increase in the NIH-HEALS total scores (p < 0.001).* |
| ***(Wang et al., 2019)*** | *To develop a communication prompt based on dignity therapy to facilitate effective conversations between patients with hematologic neoplasms and their family caregivers and to improve the program and preliminarily explore the benefits and challenges of family participatory dignity therapy (FPDT).* | *Multiple methods (single-group pre-test post-test and qualitative)* | *N=10 patient-family dyads. Patient demographics: - Mean age: 44.60 (SD 14.41 years, range=28-68) - Race/ethnicity: Chinese (100%)*  *- Sex: Female (40%); Male (60%) - Diagnosis: Acute Leukemia (60%), non-Hodgkin Lymphoma (20%), Multiple Myeloma (10%), Myelodysplastic Syndrome (10%)* | *Intervention: Family participatory dignity therapy (FPDT) - four session protocol. Session 1: introduction to FPDT. Session 2: voice-recorded interviews, each lasting 45-60 minutes. Two or three interviews were conducted to complete the intervention. The interviews were subsequently transcribed by a therapist to produce a document version. A vivid e-product called "Confessions of Love" was made for each pair of participants, containing words from interviews, photos and music chosen by the pair. Session 3: reviewing interview transcripts and collecting photos. Session 4 was sharing the final product and gathering feedback.*  *Control: N/A* | *Spiritual well-being increased from 30.30 ± 3.65 to 38.80 ± 7.29(t = 4.13, p = .003). Qualitative data indicated that the project was meaningful and well received.* |
| ***(Xiao et al., 2022)*** | *To examine the effectiveness of family-oriented dignity therapy in improving dignity-related distress, depression, and spiritual well-being in Chinese patients with lung cancer undergoing chemotherapy.* | *RCT* | *Total N=120 dyads of patients and their family caregivers. Patient Demographics: - Mean age: 56.03 (SD=8.79, range=32-73) - Race/ethnicity: Chinese (100%)*  *- Sex: Female (32.5%); Male (67.5%) Diagnosis/Cancer Type: Small cell Lung Cancer (23.3%), Non-small Cell Lung Cancer (76.7%) - Stage: 2 (5.8%); 3 (30.8%); 4 (57.5%), Limited (1.7%); Extensive (4.2%)* | *Intervention: Family-oriented dignity therapy (face-to-face psychosocial intervention with three sessions delivered by the intervention facilitator). Session 1: introduction to the intervention process and the question protocol. Session 2: Patients and family caregivers gathered to speak on the same questions to promote their communication and expression. Sessions were recorded and transcribed and the text was edited and collated into a Spiritual Diary, typically consisting of the following themes: "my family," "memorable stories," "important roles," "proudest achievement," "life lessons," "hopes and dreams," "things I want to share with my family," and "family messages." A colored version with photographs was given to patients and families. Session 3: Patients and family caregivers gathered to read the Spiritual Diary and share their feelings and thoughts regarding the reminiscence process and the diary.   Control: Apart from the usual care offered by the hospital, the patients in this group received three attention contacts according to a similar schedule to those in the intervention group.* | *The intervention group showed significantly greater improvement in spiritual well-being at both T1 (after completion of the intervention) and T2 (week four post-intervention) with respect to T0 than the control group. Significant differences between the changes in the two groups were observed in meaning level at T1 (p = 0.002), and in faith level at both T1 (p = 0.012) and T2 (p = 0.007).* |
| ***(Yun et al., 2017)*** | *To compare and examine the effectiveness of mind subtraction meditation (MSM) and a self-management education (SME) group on breast cancer survivors.* | *RCT* | *N=52  - Mean age: 48.4 (SD=8.2) - Race/ethnicity: South Korean (100%)*  *- Sex: Female (100%) Diagnosis/Cancer Type: Breast (100%) - Stage: 1 (42.3%), 2-3(57.7%)* | *Intervention: Mind subtraction meditation (MSM) - MSM twice a week, 2hr per session, for 8 weeks (16 sessions total). Instructions for the sessions were provided by 1 main and 3 assistant instructors certified in MSM. Participants were given 4 sessions, 1hr each, of SME during the first 2 weeks, which included educative sessions on understanding life after cancer treatments, an exercise regimen for health, dietary management, and follow-up surveillance. The full-scale meditation began at the fifth session. Self-directed discovery of the most difficult aspects of oneself and the evaluation of difficulty level in numeric scales; this was to specifically evaluate the negative mindsets to eliminate. Once participants identified difficult mindsets (worry about disease, fear of recurrence, sadness, sorrow, conflicts in relationships, hatred, anger, obsession, and negative self-image), they were guided to subtract these mindsets through meditation. Participants were also sent texts and e-mails encouraging home meditation 2 times each week.  Control: Self-management education (SME): Education sessions administered primarily in a lecture format, once per week for 2hr each session over 4 weeks. Contents were equal to the SME content the MSM group received during the first 4 sessions. Lectures on relationship improvement, communication skills, enhancing comfort and managing stress, and breast self-examinations were also added. Classes were instructed by 3 certified oncology nurses, an oncologist, a dietitian, and a professional coach.* | *There were significant group time effects shown in posttraumatic growth, and satisfaction with life scores No significant group differences between pre- and post-week tests. However, between 4-week and 8-week post-tests, there were very significant group differences (P<.001; P<.001; P=.007)* |
| ***(Zernicke et al., 2014)*** | *To investigate the feasibility and impact of an online synchronous Mindfulness-Based Cancer Recovery (MBCR) group program for underserved distressed cancer survivors.* | *RCT* | *N=62 total participants - Mean age: 58 (range=29-79) - Race/ethnicity: White (92%)*  *- Sex: Female (72.6%); Male (27.4%) Diagnosis/Cancer Type: Breast (33.9%); Blood/Lymph (11.3%); Colon/Gastrointestinal (11.3%); Prostate (6.4%); Female Genitourinary (9.7%); Thyroid (6.4%); Other (21%) - Stage: 1 (33.9%); 2 (24.2%); 3 (14.5%); 4 (14.5%) NR (12.9%)* | *Intervention: Online MBCR. Components of the online MBCR program were modeled after the F2F MBCR program. A licensed clinician with experience in teaching online MBSR led all MBCR intervention groups. Programs consisted of weekly 2hr sessions for 8 weeks. Didactic instruction, experiential practice, and group process were emphasized, as well as opportunity for extended practice during an online 6hr retreat between weeks 6-7. Guided meditation recordings and videos were distributed to support the home practice of 45min of Hatha yoga and mindfulness mediation daily. During online class sessions, the instructor guided experiential activities of Hatha yoga intended as "mindful movement," Qi-gong mindful movement, and as sitting, walking, and loving-kindness meditations. The instructor encouraged communication and support within the online environment to enhance group process. Headsets, webcams, and MBCR manuals were provided.   Control: TAU Wait-List Control Condition - In parallel with the online MBCR intervention group, the TAU condition group completed preassessment (T1) and post assessment (T2) online measures before and after their wait period. After the wait period, the TAU control group completed the online MBCR intervention and a post-MBCR intervention assessment (T3).* | *Analyses of the FACIT-Sp total score revealed a time by group interaction. Compared with the control condition, spirituality scores increased from preintervention to postintervention for the MBCR treatment group. Cohen d effect size was 0.37 (small). Increase in spiritual well-being in MBSR intervention group compared to control.* |
| ***(Zernicke et al., 2016)*** | *To assess the effects of participation in online Mindfulness-Based Cancer Recovery (MBCR) on patient-reported outcomes in cancer patients.* | *Quasi-experimental* | *N=62 - Mean age: 57.56 (SD=10.79) - Sex: Female (72.6%); Male (24.4%) - Diagnosis/Cancer Type: Breast (33.9%); Blood/lymph (11.3%); Colon/Gastrointestinal (11.3%); Prostate (6.5%); Female Genitourinary (9.7%); Thyroid (6.5%); Other (21.0%) - Stage: 1 (33.9%); 2 (24.2%); 3 (14.5%); 4 (14.5%); NR (13.0%)* | *The original study was conducted as a randomized wait-list controlled trial.*  *Intervention: started the program within 2 weeks of randomization, while the control group waited until the next available program. The methods and results of the randomized portion of the eCALM trial comparing intervention and wait-list control groups on primary outcomes have been previously published (Zernicke et al. 2013, 2014).*  *Weekly 2hr sessions for eight consecutive weeks conducted online in a synchronous real-time format. Experiential practice, didactic instruction, and group process were emphasized components, and the program included an online 6hr weekend retreat. Videos and guided meditation recordings were provided to participants to facilitate the recommended 45 min daily home practice. Guided mindful movement activities (Hatha yoga, qigong, walking meditations) and various sitting, and compassion meditations were facilitated by the instructor in the online environment.*  *Control: Wait-list control* | *Spirituality total scores revealed a time by age interaction, which indicated a greater increase in spirituality following the online intervention among younger participants compared to older participants.* |
| ***(Zhang et al., 2019)*** | *To evaluate the feasibility and effects of the WeChat-based life review program on anxiety, depression, self-transcendence, meaning in life and hope among cancer patients.* | *Quasi-experimental* | *N=86 Mean age 59.2 years (SD=11.5, range 23-80 years)*  *Sex: Female (26.7%); Male (73.3%) Diagnosis/Cancer Type: GI (69.77%); Respiratory (19.88%), Urinary reproductive (6.98%), Other (3.48%) Staging: 4 (83.70%)* | *Intervention: WeChat-based life review program (WBLRP) plus routine care. WBLRP is an online platform which consisted of four-section e-life review interviews and four life review modules. Interviews covered participants’ entire life, including the present (cancer experience), adulthood, childhood and adolescence and a summary of their life. Memory prompts helped to evoke patients' memories and facilitate life review process by means of images, music, videos and audio-picture books. Review Extraction referred to a summary of the meaningful events where patients can view and leave their comments; Mind Space provided an opportunity to express emotions, hand down wishes, or reveal their true feelings to anyone who is important at that stage. E-legacy product was a video booklet that can be preserved as a spiritual memorial. The WBLRP was conducted weekly over 6 weeks. Each life review interview ranged from 40-60 min, depending on the patient's physical condition and willingness to talk.  Control: The control group received only routine care, which included personal care, medical care, health education and emotional support, all provided by the study hospital. The control group participants could use the Internet freely to search for information. They had no access to the WBLRP.* | *Significant improvements in self-transcendence were detected. Trends of improvement in meaning in life and hope, not significant between the two groups.* |
